# New Freedom through Medical Devices Based on the Global System for Mobile Communications: A Prospective Survey of 620 Users of the Swiss Limmex Emergency Wristwatch—An Original Study from Switzerland

**DOI:** 10.1155/2013/563731

**Published:** 2013-08-25

**Authors:** Malek Tabbara, Onur S. Özgüler, Aristomenis K. Exadaktylos

**Affiliations:** Department of Emergency Medicine, Inselspital, Bern University Hospital, 3010 Berne, Switzerland

## Abstract

About 500,000 elderly people in Switzerland suffer a fall each year. Thus medical attention and help are essential for these people, who mostly live alone without a caregiver. Only 3% of people aged over 65 in Switzerland use an emergency system. Personal telehealth devices allow patients to receive enough information about the appropriate treatment, as well as followup with their doctors and reports of any emergency, in the absence of any caregiver. This increases their quality of life in a cost-effective fashion. “Limmex”—a new medical emergency watch—was launched in Switzerland in 2011 and has been a great commercial success. In this paper, we give a brief review of this watch technology, along with the results of a survey of 620 users conducted by the Department of Emergency Medicine in Bern.

## 1. Introduction

**Figure 1:**
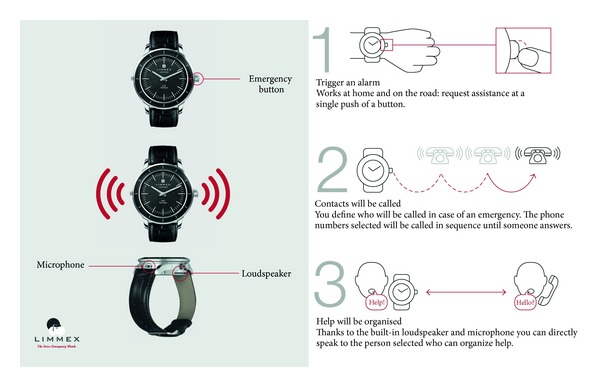
MEW, escalating alarm mechanism.

As a consequence of several demographic and social factors, the proportion of people living alone has been continuously increasing in recent decades. In urban areas such as the Bern region, about 50% of retired people live alone in single person households. The number of elderly people is increasing in Switzerland (1.4 million over 65 years), and for the great majority of these, it is very important to be able to live independently at home for as long as possible. Consequently, it is crucial that these elderly people should receive prompt medical attention and help when an emergency occurs. Studies have shown that when the elderly fall they are out of reach of the fixed-line network or a mobile phone. This is why it is important to wear an emergency system on the wrist or round the neck. Personal telehealth devices allow patients to receive enough information about the appropriate treatment, as well as followup with their doctors and reports of any emergency. This can take place in the absence of any caregiver and increases their quality of life in a cost-effective fashion [1–3]. We have found that only 37% of subjects thought that they would be found within 30 minutes of a serious emergency [4]. Despite these facts, only 3% of elderly individuals have access to a personal emergency system. In our opinion, the main reason for this is that current systems are bulky and indiscreet, which will somehow label and stigmatize their users as “sick.” In addition, some systems are complicated to install and are not fully portable. In 2011, the Swiss company “Limmex AG” and the Centre Suisse d' Electronique et de Microtechnique (Swiss Center for Electronics and Microtechnology) launched an “elegant” Swiss-made medical emergency watch (MEW) with an integrated emergency system function [5, 6]. MEW uses GSM-based technology packaged in a wristwatch. By pushing a button, the wearer can initiate multiple emergency calls and establish mobile communication with a preselected person, institution, or a search and rescue service (Figure 1). MEW is waterproof against splashes and thus can be worn when taking a shower or bath. The watch has been a great commercial success, due to its simple, reliable, and easy-to-wear design. This paper will focus on the results of a survey of 620 MEW users conducted by the Department of Emergency Medicine in Bern.

## 2. Material and Methods 

In collaboration with Limmex, the Department of Emergency Medicine of Bern University Hospital carried out an anonymous survey of MEW users (*n* = 620). The survey was conducted 18 months after the market launch of the watch and aimed to collect the initial impressions of the users of the emergency watch. The survey included questions on demographics data, length of time since first wearing MEW, wearer satisfaction, impact of the device on their daily life, and the ease of use of the watch. Descriptive statistics were used to report sample characteristics.

## 3. Results

### 3.1. Demographic Data and Basic Information

**Figure 2:**
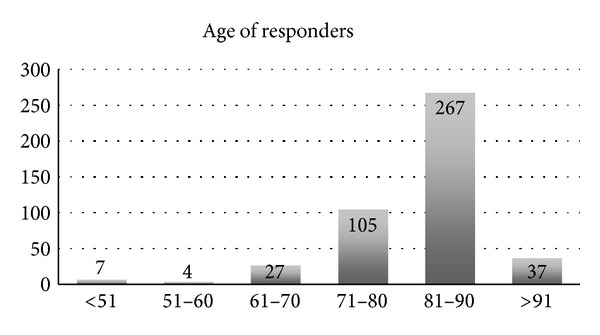
Age of responders to the survey.

**Figure 3:**
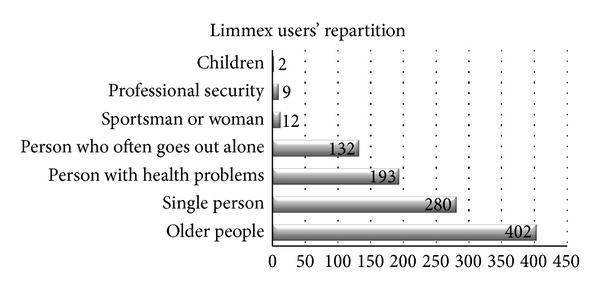
Distribution of lime users (repartition is a totally obscure word).

**Figure 4:**
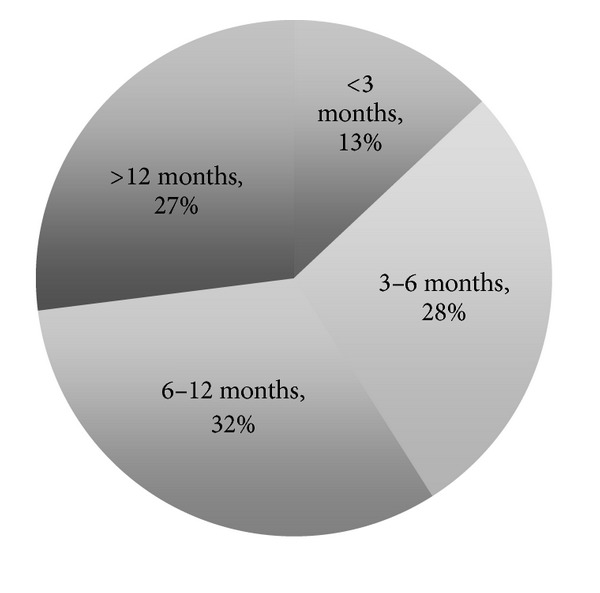
Period of wearing MEW at the time of the survey.

The survey was sent to 1,350 persons in the medical/senior segment, with a response rate of 46% (620 persons). The mean age of the respondents was 81.8 years, 66% of whom were older than 70 (Figure 2). The majority of the survey respondents were women (81%). Eighty-five percent (85%) of the respondents answered that they live alone, and only 11% stated that they lived in a 2-person household. Most respondents thought that they would need MEW because they were “older people” (402 persons, 65%), followed by “single people” living alone (280 persons, 45%) (Figure 3). Twenty-seven percent (27%) of the respondents had been using their MEW for more than a year when they took the survey (Figure 4).

### 3.2. Satisfaction and Security

Ninety-four percent (506 persons, 94%) of the respondents stated that they were satisfied or very satisfied with MEW, while only 32 (6%) respondents stated that they were dissatisfied, and 82 (13%) respondents did not answer this question. Ninety-nine percent (565 persons, 99%) stated that they would recommend it to others. The majority of the users (532 persons, 98%) felt that they were more secure in their everyday life thanks to MEW.

### 3.3. Using MEW

When asked: “When do you use MEW and during which activities?” 96% (551 persons) answered that they thought that it is important to wear it at home; 89% (481 persons) also wore it during excursions, particularly when walking or hiking, followed by working around the house (307 persons, 83%). Ninety-three percent (479 persons, 93%) of MEW users wore the watch at night and 98% (539 persons) during the day.

### 3.4. Emergency Call Trigger

**Figure 5:**
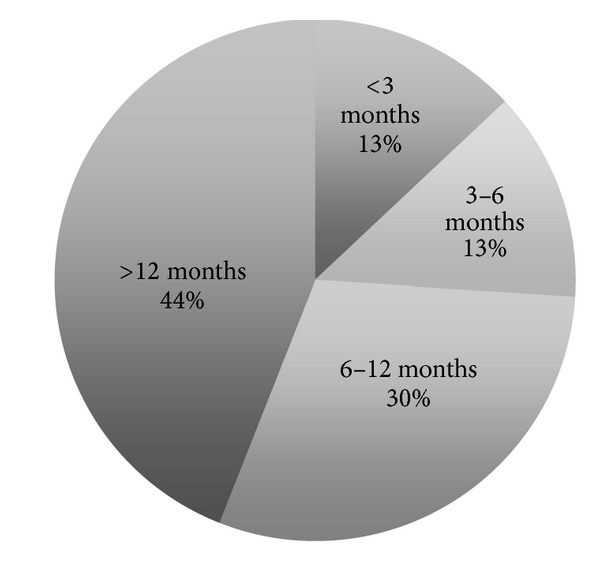
Lapse of time wearing MEW when the emergency occurred.

Ten percent (10%) of MEW wearers (55 persons) used it to call for help during an emergency. Forty-four percent of them (23 persons, 44%) had been wearing MEW for more than a year when an emergency occurred (Figure 5). The mean age of the wearer was then 81.6 years. The cause of the emergency was mostly a fall at home (38 persons, 69%).

## 4. Discussion

New research in many cultural settings is showing that older people prefer to be in their own homes and communities, even if that means living alone. This preference is reinforced by greater longevity, expanded social benefits, increased home ownership, more appropriate housing, and an emphasis in many nations on community care [7]. Researchers at Geneva and Lausanne universities have carried out a study analysing data on the demographic change, which was collected during the Swiss Census in 2000; this also supported this trend. They concluded that old people in Switzerland are living longer, healthier, and more independently, and that since 1990, the proportion of old people living in nursing homes has gone down [8]. Our study confirms this trend; the mean age of persons responding to the survey was 82 years, with the majority of them (85%) stating that they lived alone, and 45% of the respondents chose this reason for seeking an emergency personal system. 

In our previous study, approximately 60% of those interviewed stated that they would feel safer if they had a device which enabled them to alarm a close person of their choice, the family doctor, or an emergency service in any medical emergency [4]. In our current survey, 98% of the users stated that they felt more secure in their everyday life thanks to MEW. Most of the users (94%) expressed their satisfaction with MEW, and 99% noted that they would recommend MEW to others. Similar results have been described by Mann et al., who surveyed 606 persons using a personal emergency response system (PERS); in their study, 76% of the participants expressed an enhanced feeling of security when using PERS [9]. During a 1-year follow-up period of 106 patients using PERS, Roush et al. found that there was a statistically significant decrease in hospital admissions per person and in time in hospital [10]. A study by Bernstein concluded that use of monitored PERS reduces mortality rates nearly fourfold and hospital utilization by 59% [11]. In the study of Mann et al., fear of falling was most often given as the reason for using a PERS in Mann et al. study [9]. In our study, a fall was found to be the main cause (69%) of emergency calls triggered using MEW. 

Our study confirms the few studies published on PERS. The limitations were due to the fact that it is based on a survey, with only 10% of patients triggering an alarm call using MEW. The next step in confirming the reliability and ease of use of MEW will be to perform a follow-up study, looking more into the experience of users who actually triggered a call to the emergency services using their watch, together with their feedback about the product. 

## 5. Conclusion

MEW has proved itself to be an innovative product; it provides a simple and reliable personal emergency system while delivering the accurate time with a well-designed Swiss-made watch. MEW was found to increase the feeling of safety and security among its users, with most of them expressing satisfaction. Doctors should introduce this system to suitable patients, particularly the elderly living alone. Patients can be referred to the Red Cross, as they can offer a variety of emergency systems, recently including MEW watches, and can provide expert information. This can effectively help in avoiding falls followed by long periods of lying on the floor, which can have serious medical consequences.
